# Multiple and Variable NHEJ-Like Genes Are Involved in Resistance to DNA Damage in *Streptomyces ambofaciens*

**DOI:** 10.3389/fmicb.2016.01901

**Published:** 2016-11-28

**Authors:** Grégory Hoff, Claire Bertrand, Lingli Zhang, Emilie Piotrowski, Ludovic Chipot, Cyril Bontemps, Fabrice Confalonieri, Stephen McGovern, François Lecointe, Annabelle Thibessard, Pierre Leblond

**Affiliations:** ^1^UMR 1128, Dynamique des Génomes et Adaptation Microbienne, Université de LorraineVandœuvre-lès-Nancy, France; ^2^UMR 1128, Institut National de la Recherche Agronomique, Dynamique des Génomes et Adaptation MicrobienneVandœuvre-lès-Nancy, France; ^3^Institute for Integrative Biology of the Cell (I2BC), CEA, Centre National de la Recherche Scientifique, Université Paris-SudOrsay, France; ^4^Institut Micalis, INRA, AgroParisTech, Université Paris-SaclayJouy-en-Josas, France

**Keywords:** *Streptomyces*, non-homologous end joining, DNA repair, double strand breaks (DSBs), ligases, Ku protein, DNA damage

## Abstract

Non-homologous end-joining (NHEJ) is a double strand break (DSB) repair pathway which does not require any homologous template and can ligate two DNA ends together. The basic bacterial NHEJ machinery involves two partners: the Ku protein, a DNA end binding protein for DSB recognition and the multifunctional LigD protein composed a ligase, a nuclease and a polymerase domain, for end processing and ligation of the broken ends. *In silico* analyses performed in the 38 sequenced genomes of *Streptomyces* species revealed the existence of a large panel of NHEJ-like genes. Indeed, *ku* genes or *ligD* domain homologues are scattered throughout the genome in multiple copies and can be distinguished in two categories: the “core” NHEJ gene set constituted of conserved loci and the “variable” NHEJ gene set constituted of NHEJ-like genes present in only a part of the species. In *Streptomyces ambofaciens* ATCC23877, not only the deletion of “core” genes but also that of “variable” genes led to an increased sensitivity to DNA damage induced by electron beam irradiation. Multiple mutants of *ku*, ligase or polymerase encoding genes showed an aggravated phenotype compared to single mutants. Biochemical assays revealed the ability of Ku-like proteins to protect and to stimulate ligation of DNA ends. RT-qPCR and GFP fusion experiments suggested that *ku*-like genes show a growth phase dependent expression profile consistent with their involvement in DNA repair during spores formation and/or germination.

## Introduction

Double strand breaks (DSB) are the most detrimental genomic lesions and their repair constitutes a major challenge for cell viability. In eukaryotes and prokaryotes, two main repair machineries have evolved to repair these DSBs, namely homologous recombination (HR) and non-homologous end joining (NHEJ) (Alonso et al., 2013; Deriano and Roth, 2013; Glickman, 2014). In contrast to HR which needs an intact template to ensure repair of the break, NHEJ can bind DNA ends together without any template after a facultative DNA ends remodeling step. In multicellular eukaryotes, the NHEJ mechanism represents the predominant DSB repair pathway and requires the intervention of the end tethering heterodimer Ku70/80 as well as diverse end processing proteins (for review: Deriano and Roth, 2013). In prokaryotes, NHEJ is not ubiquitous and the minimal NHEJ machinery first described in *Mycobacterium* consists of two actors: a Ku homolog and an ATP dependent ligase LigD (Weller et al., 2002; Della et al., 2004; Gong et al., 2005). The latter one is a multifunctional protein that carries three distinct enzymatic domains conferring nuclease, polymerase and ligase activities (Pitcher et al., 2005). Unlike DSB repair through HR, DSB repair carried out by NHEJ is mutagenic. Nucleotide addition and/or deletion are scars frequently left after DNA ends ligation (Stephanou et al., 2007; Aniukwu et al., 2008; Gupta et al., 2011). The initial model involving two unique actors is now renewed by the discovery of alternative actors. When the ligase domain of LigD (LigDom) is defective, a back-up ligation activity is provided by LigC, another ATP dependent ligase (Della et al., 2004; Bhattarai et al., 2014). In the same way, *Mycobacterium smegmatis* possesses two additional polymerases which share the same *in vitro* activity than the polymerase domain of LigD (PolDom) (Zhu et al., 2012).

*Streptomyces* are filamentous bacteria mainly found in soils, sediments and seawater as well as symbionts with plants, fungi and animals (Seipke et al., 2012). Soil is a complex and changing environment where *Streptomyces* have to adapt to the heterogeneousness of the niche (moisture, nutrients, temperature…) and to cope with biotic competition. Their rich and varied secondary metabolism such as signaling molecules or chemical weapons is assumed to mediate their relationships with other organisms (Davies and Davies, 2010; Abrudan et al., 2015). Under limited nutritive conditions, their hyphae differentiate into long chains of exospores providing an efficient way for resistance and dissemination until suitable conditions for another growth cycle are met (Chater, 1993). While hyphae are large multi-genomic compartments, spores are known to include a single genome (Hopwood, 2006). In contrast to the vast majority of bacteria, *Streptomyces* possess a linear chromosome whose size is ranging from 5.96 to 11.94 Mb ^1^ with a central replication origin and terminally inverted repeats that are covalently bound to specific telomere proteins (Lin et al., 1993). The *Streptomyces* genome presents a high plasticity and their chromosome is highly compartmented, with a conserved central region including mostly housekeeping genes whereas the dispensable genes are predominantly located in the chromosomal arms (Bentley et al., 2002; Ikeda et al., 2003; Kim et al., 2015). The loss of synteny at the ends of the chromosome results from horizontally acquired DNA insertion or deletion events (Choulet et al., 2006; Thibessard and Leblond, 2014). This terminal variability reveals the strong recombination activity taking place at the ends of the genome which is reminiscent of the DNA rearrangements reported several decades ago (Leblond et al., 1990; Volff and Altenbuchner, 1998). Hence, *Streptomyces* are also subject to a high level of genetic instability which is directly linked to major genome rearrangements. Characterization of some of these rearrangements revealed large deletions, chromosome circularization, (Fischer et al., 1997; Inoue et al., 2003), arm replacement (Fischer et al., 1998; Uchida et al., 2003), chromosome end-to-end fusion (Wenner et al., 2003) or large DNA amplifications (Yanai et al., 2006). Molecular analyses showed that chromosome circularization and end-to-end fusion events especially rely on illegitimate recombination whereas chromosome arm replacement or DNA amplifications is dependent on HR. This terminal recombination pattern may result from higher recombination frequencies triggered by the preferential formation of DSBs in these regions. On the other hand, genome rearrangements could also be more tolerated if formed in these dispensable regions.

Nevertheless, little is known about DSB repair in *Streptomyces*. Like the other bacteria, *Streptomyces* possess a RecA homologue involved in HR (Aigle et al., 1997; Muth et al., 1997; Vierling et al., 2001; Huang and Chen, 2006). However, recent studies that we performed suggest that *Streptomyces* has an atypical HR pathway. In fact, in contrast to bacterial models like *Escherichia coli, Bacillus subtilis* or *Mycobacterium*, deletion of genes encoding a potential helicase-nuclease complex supposed to be involved in the initial end resection step during HR is not viable in *S. ambofaciens* (Zhang et al., 2014). On the other hand, deletion of *ruvABC* and *recG*, two loci encoding factors that have a redundant role in migration and resolution of Holliday junction during late steps of HR, has only a mild effect on recombination efficiency (Hoff et al., 2016). Early bioinformatics studies showed that the two model species, *S. coelicolor* and *S. avermitilis*, both possess a *ku* ortholog suggesting also the existence of a NHEJ pathway in *Streptomyces* (Rocha et al., 2005). Here, we performed *in silico* analyses in the fully sequenced *Streptomyces* genomes leading to the identification of a large panel of conserved and variable genes suspected to be involved in a NHEJ pathway. Construction of single and multiple NHEJ-like genes mutant strains in *S. ambofaciens* allowed us to study the impact of these different genes in DNA damage repair. All the results we obtained suggest the existence of a complex and atypical bacterial NHEJ pathway in *Streptomyces*.

## Materials and Methods

### Phylogenetic Analyses

A total of 38 sequenced *Streptomyces* genomes, including that of *S. ambofaciens* ATCC23877 was scanned to identify genes putatively encoding NHEJ Ku-like or LigD-like proteins based on BLAST similarity search against the non-redundant database. A preliminary BLASTP search (*e*-value cut-off 10^-30^_;_ identity threshold 30%) using *M. tuberculosis* Rv0937c and Rv0938 (Ku and LigD respectively) protein sequences as a query allow to identify and numerate the candidates. Secondly, BLASTP were performed using *S. ambofaciens* ATCC23877 NHEJ-like genes and reciprocal best hits allowed to organize them into groups of orthologs.

For the phylogeny of Ku-like proteins, the tree was built using a maximum-likelihood method based on a GTR + G + I model. Phylogenetic trees were built and edited with Mega6 (Tamura et al., 2013). Support of the tree branches was estimated with 100 bootstrap replicates and all positions with <80% site coverage were eliminated. A total of 295 and 309 positions were respectively used for the Ku-like and the LigC/LigD protein phylogenetic trees.

### Bacterial Strains and Plasmids

All strains used in this study are listed in **Table 1**. The DH5α *E. coli* strain is used as conservation host for BACs and plasmids. For biochemical analyses, plasmid constructions were done *in E.coli* MC1061 (Casadaban and Cohen, 1980). ER2566 (NEB) was used for *kuB* gene expression and Rosetta (DE3) PLysS (Stratagene) for *kuA* and *kuC* gene expression. *E.coli* strains were grown in Luria-Bertani (LB) medium at 37°C except for the BW25113 pKD20 thermosensitive strain used for PCR targeting, which was grown at 30°C. *S. ambofaciens* strains were grown at 30°C on Soya flour mannitol (SFM) plates except for phenotypical analyses and for liquid cultures which were performed on solid Hickey Tresner (HT) medium. All *Streptomyces* mutants derive from our reference strain *S. ambofaciens* ATCC23877 (Pinnert-Sindico, 1954). Mutant strains were constructed by PCR targeting as described by Gust et al. (2003). To summarize, a recombinant BAC containing the locus of interest was transformed in the highly recombinogenic *E. coli* BW25113/pKD20 strain in order to replace the target gene by an *oriT*- *aac(3)IV* resistance disruption cassette (Raynal et al., 2006). The ET12567 non-methylating strain containing the mobilizing pUZ8002 plasmid was used as donor for intergeneric conjugation of the modified BAC between *E. coli* and *S. ambofaciens* Double-crossover (CO) events leading to the replacement of the target gene by the cassette were selected by the sensitivity to kanamycin (loss of the BAC vector part) along with the resistance to apramycin (insertion of the resistance cassette). The disruption cassette flanked by *attL* and *attR* sites was excised in the newly obtained mutant strains thanks to the introduction of pOSV508 a plasmid allowing the expression of *int* and *xis* genes from pSAM2 (Raynal et al., 2006). Strains expressing translational EGFP fusions were also constructed by *in vivo* recombination. The *egfp* gene with the transcription terminator from phage λ was cloned from plasmid pIJ8660 (Sun et al., 1999) into an *oriT aac(3)IV* disruption cassette (Raynal et al., 2006). This cassette was then used as the source to make *kuA-egfp, kuB-egfp* and *kuC-egfp* fusions. PCR was performed using *egfp-oriT-aac(3)IV* as the template with specific oligonucleotides which anneal to the linker sequence at the 5′ end of *egfp* and downstream of the 3′ end of *aac(3)IV* and introduce 40 bases of homology to the 3′ ends of *kuA, kuB*, or *kuC*, as well as removing the stop codons. PCR products were introduced into *E. coli* BW25113/pKD20 containing the cognate BACs with either *kuA, kuB*, or *kuC*, and the following steps of the procedure are described above. In these recombinant strains, the fusion genes (*kuA-egfp, kuB-egfp* or *kuC-egfp*, respectively) are the only copies of *kuA, kuB*, or *kuC*.

**Table 1:** *Streptomyces ambofaciens* strains used in this study.

Strains	Characteristics	Sources
ATCC23877	Used as a WT strain	Pinnert-Sindico, 1954
*ΔkuA*	*ΔSAM23877_5082*	This study
*ΔkuB*	*ΔSAM23877_6929*	This study
*ΔkuC*	*ΔSAM23877_6942*	This study
*ΔkuABC*	*ΔSAM23877_5082 ΔSAM23877_6929 ΔSAM23877_6942*	This study
*ΔligC*	*ΔSAM23877_6361*	This study
*ΔligD*	*ΔSAM23877_0862*	This study
*ΔligCD*	*ΔSAM23877_6361 ΔSAM23877_0862*	This study
*ΔpolR*	*ΔSAM23877_6362*	This study
*ΔpolK*	*ΔSAM23877_5081*	This study
*ΔpolRK*	*ΔSAM23877_6362 ΔSAM23877_5081*	This study
*ΔrecA*	*ΔSAM23877_5473*	This study
*ΔkuABC ArecA*	*ΔSAM23877_5082 ΔSAM23877_6929 ΔSAM23877_6942 ΔSAM23877_5473*	This study

### Electron Beam Irradiation (EBI)

An amount of 10^9^ spores of different *Streptomyces* strains was diluted in sterile water in 96 well microplates and exposed to 0, 0.1, 0.3, 0.5, 0.7, and 1 kGy of radiation doses. Processing was carried out in a Van de Graaf^®^ electron accelerator (VIVIRAD, Handschuheim, France) in the technological resource center Aérial (Illkirch-Graffenstaden, France). Energy delivered by the electron accelerator reach 2.2 MeV with an intensity of 10 μA. Dose rate equals approximately 60 kGy/h. After irradiation, several spore dilutions were plated with the easySpiral^®^ apparatus (Interscience, Saint-Nom-la-Bretèche, France) on SFM Petri dishes and the number of colonies was counted after 3 days incubation at 30°C. For each genetic context, at least two independent mutant strains were exposed in two independent experiences.

### Fluorescent Microscopy

Strains for microscopic observations were inoculated in the acute-angle junction of standard-sized microscope coverslips inserted with an angle of 45° in SFM agar, and grown for 48 h. The coverslips were then carefully pulled from the medium and directly mounted in a drop of sterile water on a microscope slide. Fluorescent microscopy observations were carried out using a Axiovert 200M MOT device (Carl Zeiss Micro imaging) equipped with a mercury vapor lamp and a FITC filter set (470/40 excitation, 540/50 emission). Both contrast phase and fluorescent observations were made with an X100 objective.

### DNA Preparation

To verify the mutant strains, *Streptomyces* genomic DNA was extracted by low binding sulfate salt method as described by Kieser et al. (2000). Liquid HT medium was used for growth of mycelium before DNA extraction. For biochemical analyses, the gene coding for KuA was amplified from the BAC 16ZB5. *KuB* and *KuC* genes were amplified from the BAC 27ZF1. Primers used for amplification were designed to flank the open reading frames with the coding sequence of a His_6_-tag followed by a *NdeI* site on their 5′ side, and by a *XhoI* site on their 3′ side. The resulting ORFs were cloned under the T7 promoter of the pJ411 vector (DNA2.0) giving the plasmids pSMG258, 259, 260 allowing expression of the proteins 6His-KuA, 6His-KuB, 6His-KuC, respectively (named hereafter KuA, KuB, and KuC). Sequences of the primers used in this study are available upon request.

### RNA Extraction and RT-qPCR Analyses

For RNA extraction from cultures on solid medium, streaks were harvested after growth (from 24 to 60 h depending of the experiment) at 30°C on HT solid medium covered with cellophanes membranes. For RNA extraction after a genotoxic stress, pre-germinated spores of *Streptomyces* were grown until 0.2 OD600 was reached and exposed to mitomycin C (MMC, 1.5 μg.mL^-1^, i.e., CMI/4) for 30 min. RNA isolation was performed using the Aurum Total RNA mini-kit (Bio-Rad) according to the manufacturer’s instructions. To optimize cell lysis, an additional sonication step (3 × 30 s) using a Bioruptor apparatus was added. The RNA quality was assessed after migration on 1% agarose gel. In order to eliminate genomic DNA contamination, the RNA samples were treated before reverse transcription with DnaseI (Ambion) in the presence of 10 U of RNase inhibitor per μg of RNA. DnaseI was then inactivated at 75°C during 10 min in the presence of EDTA. Reverse transcription was performed on 2 μg RNA with an iScript Advanced cDNA synthesis kit (Bio-Rad) according to the manufacturer’s instructions. The absence of contamination with genomic DNA was validated by a 30 cycle-PCR on a control aliquot removed before reverse transcription using hrdB-F and hrdB-R primers. Quantitative PCR was undertaken in a CFX96 (Bio-Rad) apparatus in a 10 μL reaction mixture containing respectively 5 μL of SYBR supermix (Bio-Rad), 0.2 μM of each primer and 4 μL of a 10-fold dilution of cDNA. The reaction conditions were as follow: a 3 min initial denaturation step at 95°C and 40 cycles of 10 s at 95°C and 30 s at 60°C. In order to check the absence of secondary products, melting curves were realized from 65 to 95°C with an increase rate of 0.5°C/5 s. The transcription level of interest genes was analyzed using the mathematical model proposed by Pfaﬄ (2001). The *hrdB* gene, encoding the principal sigma factor in *Streptomyces*, was used as the reference gene for the normalization of the expression level as it was the case in former studies (Galet et al., 2015; Martínez-Burgo et al., 2015).

### Proteins Purification

#### Purification of KuA

*Escherichia coli* Rosetta PlysS cells transformed with pSMG258 were grown at 30°C in 2 l of LB medium supplemented with 25 mg/mL thymine, 30 μg/mL kanamycin and 25 μg/mL chloramphenicol to OD600 = 0.7. Production of protein was induced with 0.5 mM IPTG for 15 h at 20°C. Cells were harvested by centrifugation and the pellet was resuspended in 40 mL lysis buffer A (50 mM Tris-HCl pH 8, 0.25 M NaCl). Cells were broken by a freeze/thaw step in liquid N_2_/37°C and then centrifuged 1 h at 100 000 *g* at 4°C. The pellet was resuspended with 40 mL of a 20 mM Tris pH 7.5, 2.5 mM MgCl_2_, 0.5 mM CaCl_2,_ 1 mg/mL DNaseI buffer. After 1 h at 20°C, the mixture was centrifuged 20 min at 100 000 *g* at 4°C. All subsequent steps were carried at 4°C. The pellet was washed with 40 ml buffer P_1_ (50 mM Tris-HCl, pH 8, 1 M NaCl), and then solubilized with 40 mL of buffer P_2_ (50 mM Tris pH 8, 8 M urea). After 1 h the mixture was centrifuged 20 min at 100 000 *g* and 3 mL of Ni^2+^ affinity column (Ni-NTA agarose, Qiagen) was added to the supernatant which was transferred onto an Econo-Column (Biorad). The flow through was discarded and the resin was washed with series of 25 mL of buffer P_3_ (50 mM Tris pH 8, 1% sucrose, 0.5 M NaCl) with urea concentration decreasing from 8 M to 0 M by steps of 0.5 M. The Ni-NTA column was washed with buffer P_3_, then with buffer W (50 mM Tris pH 8.0, 0.5 M NaCl) supplemented with 20 mM imidazole. KuA protein was eluted in buffer W supplemented with 250 mM imidazole and then dialysed against buffer D (50 mM Tris-HCl pH 8, 0.4 M NaCl, 50% glycerol, 1 mM DTT) prior to storage at -20°C.

#### Purification of *KuC*

The expression of *KuC* gene was induced in *E. coli* Rosetta PLysS cells transformed with pSMG260 as described for KuA. Cells were harvested by centrifugation and the pellet was resuspended in 40 mL lysis buffer B (50 mM Tris-HCl pH 8, 0.25 M NaCl, 0.2 mg/mL lysozyme) supplemented with Complete Antiprotease (Roche) and incubated 1 h at 20°C. The mixture was then centrifuged and the pellet was treated with DnaseI, as described for KuA purification. Purification procedure of KuC at this step is the same as the one described for KuA.

#### Purification of KuB

ER2566 cells transformed with pSMG259 were grown at 30°C in 2 l of LB medium supplemented with 25 mg/mL thymine and 30 μg/mL kanamycin and *kuB* gene expression was induced as for *kuA* and *kuC* genes. Cells were harvested by centrifugation, the pellet was resuspended in 40 mL of lysis buffer B and incubated 1 h at 20°C. All subsequent steps were carried at 4°C. Cell lysis mixture was supplemented with 0.5 M NaCl, 0.1% Triton and 0.1% Non-idet P40 and incubated 1 h. Two mL of polyethyleneimine (PEI) 10%, pH 8 was added to precipitate nucleic acids; the mixture was stirred for 1 h and then centrifuged 1 h at 100 000 *g*. The soluble phase was next precipitated by raising the concentration of ammonium sulfate (AS) firstly to 50% of saturation, KuB was mainly found in the supernatant at this step, and secondly to 80% of saturation to precipitate KuB. The corresponding pellet was resuspended in 25 mL of buffer P_1_ and 3 mL of Ni-NTA agarose, pre-equilibrated in buffer P_1_, were added. The Ni-NTA agarose was washed with buffer P_1_, then with buffer W supplemented with 20 mM imidazole and KuB was eluted in buffer W supplemented with 250 mM imidazole. The eluted fraction was dialyzed against buffer D and stored at -20°C.

Final purification step analyzed by SDS PAGE showed that purified KuA, KuB and KuC were obtained at 90% purity and were a mix of full length and truncated forms (Supplementary Figure S1). Their concentration was determined by UV absorption at λ = 280 nm on a NanoDrop 1000 Spectrophotometer (Thermo Scientific).

#### Purification of LigD_Bsub_

LigD from *Bacillus subtilis* fused in C-terminal to a His_6_ tag was purified as previously described McGovern et al. (2016).

### T5 Protection Assay

Fifty nanograms (∼75 fmol) of a 1001 bp linear DNA substrate with 5′-phosphorylated blunt-ends were preincubated with raising concentrations of KuA, KuB, or KuC in 19 μL of buffer T5 (50 mM Tris-HCl pH 8, 1 mM DTT, 5 mM MgCl_2_, 40 mM NaCl and 5% glycerol) for 30 min at 37°C. One μL of T5 exonuclease (0.5 U/μL, Biolabs) was added to the reaction mixture and reactions were incubated for 30 min at 37°C. Reactions were stopped by adding 4 μL of stop buffer (5 mg/mL proteinase K, 2% SDS and 0.1 M EDTA) and incubated at 37°C for 30 min. Four microliter of DNA loading buffer (33% glycerol and 0.25% xylene cyanol) were added, and the reactions were loaded on a 0.7% agarose gel in TBE Buffer. After electrophoresis, DNA was stained by the SYBR Gold reagent (Invitrogen) and the gel was photographed using a Chemidoc apparatus (Bio-Rad).

### Ligation Assay

In a final reaction volume of 20 μL in the T4 DNA ligase ligation buffer (NEB, 50 mM Tris-HCl pH 7.5, 10 mM MgCl_2_, 10 mM DTT, 1 mM ATP), 50 nanograms (∼75 fmol) of the 1001 bp linear DNA substrate were preincubated with 0.25 or 1 μM of KuA, KuB, or KuC for 30 min at 30°C. Then, raising concentrations of LigD*_Bsub_* were added to reactions. Ligation reactions were stopped after 2 h at 37°C by adding 4 μL of stop buffer and analyzed as described above.

## Results

### A “Core” and a “Variable” NHEJ Gene Set in *Streptomyces*

**FIGURE 1:**
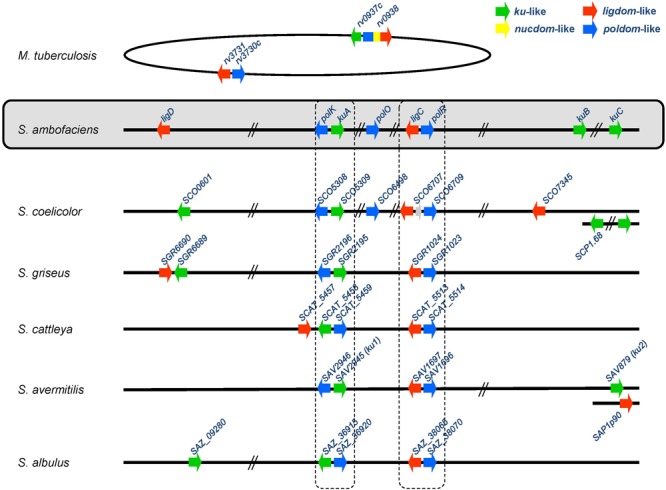
**Organization of the NHEJ-like genes in several *Streptomyces* genomes.** The two conserved loci *kuA-polK* and *ligC-polR* are framed in dotted lines.

Genes homologous to the bacterial NHEJ factors *ku* and *ligD* were sought in the genome of our model strain *Streptomyces ambofaciens* ATCC23877 (Thibessard et al., 2015). To do so, the *M. tuberculosis* Rv0937c and Rv0938 (Ku*_Mtub_* and LigD*_Mtub_* respectively) protein sequences were used as query in a BLASTP search. The first observation was that the LigD*_Mtub_* protein had no full length homologue in *S. ambofaciens*, but that each candidate protein matched only with one single domain at a time. Therefore, the 759 amino acid sequence of LigD*_Mtub_* was split into 3 slightly overlapping parts (1–300, 271–459, and 451–759) corresponding to the polymerase (PolDom), the nuclease (NucDom) and the ligase (LigDom) domains, respectively. In this way, 3 Ku-like encoding genes (sharing 36, 39, and 40% of protein identity with Ku*_Mtub_*), 3 LigDom-like encoding genes (sharing 30, 32, and 41% of protein identity with the LigDom of LigD*_Mtub_*) and 3 PolDom-like encoding genes (sharing 29, 34, and 41% of protein identity with the PolDom of LigD*_Mtub_*) were identified in *S. ambofaciens* ATCC23877 genome (**Figure 1**). No NucDom-like encoding gene was detected. The 3 *ku*-like genes (*SAM23877_5082, SAM23877_6929* and *SAM23877_6942*) were named *kuA, kuB*, and *kuC* respectively. Two of the 3 LigDom-like genes (*SAM23877_6361* and *SAM23877_0862*) were named *ligC* and *ligD*, respectively. Indeed, *SAM23877_6361* appeared to share more amino-acid identity to *M. tuberculosis* LigC (LigC*_Mtub_*), i.e., 60% than to the LigDom of LigD*_Mtub_* i.e., 30% and was therefore considered as LigC*_Mtub_* ortholog. Similarly, *SAM23877_0862* appeared to be the closest relative of the LigDom of LigD*_Mtub_*. The third ATP-dependent DNA ligase encoding gene detected (*SAM23877_1283*) would encode a LigB protein known to exhibit a strong nick sealing activity *in vitro* and whose defect would not alter mycobacterial NHEJ (Gong et al., 2004). Therefore, this third ligase was not included in our further investigations. The 3 PolDom-like genes (*SAM23877_5081, SAM23877_6202*, and *SAM23877_6362*) were named *polK, polO* and *polR*, respectively. It was noticeable that 2 of the 3 PolDom like genes (*polK* and *polR*) colocalized with *kuA* and *ligC*.

KuA, KuB, and KuC proteins are composed of 365, 383, and 302 amino-acids, respectively. All these proteins harbor the conserved Ku core domain at their N-terminal side, the conserved minimal C-ter domain (red in Supplementary Figure S2), that has been shown to be required for the interaction with LigD in *B. subtilis* (McGovern et al., 2016), and a basic extended C-terminal domain, which is of 114, 128, and 47 aa, respectively. The pIs of these extended domains (11.64, 10.97, and 10.65) are consistent with those described for the majority of bacterial Ku proteins. This suggests that their roles in DNA binding as well as in the modulation of the ability to thread inward the DNA molecule are conserved in *Streptomyces* (Kushwaha and Grove, 2013; McGovern et al., 2016). Interestingly, a SAP domain, previously predicted in the *S. coelicolor* KuB homologue (*SCO0601*) by Aravind and Koonin (2001) is also detected in *S. ambofaciens* KuB (at position 337–370, Supplementary Figure S2). This SAP domain is assumed to be also involved in nucleic acid binding (Aravind et al., 2000) and could modify the DNA binding abilities of KuB compare to KuA and KuC. Further experiments are required to highlight the roles and the differences of the extended C-terminal domains of these three Ku proteins.

**FIGURE 2:**
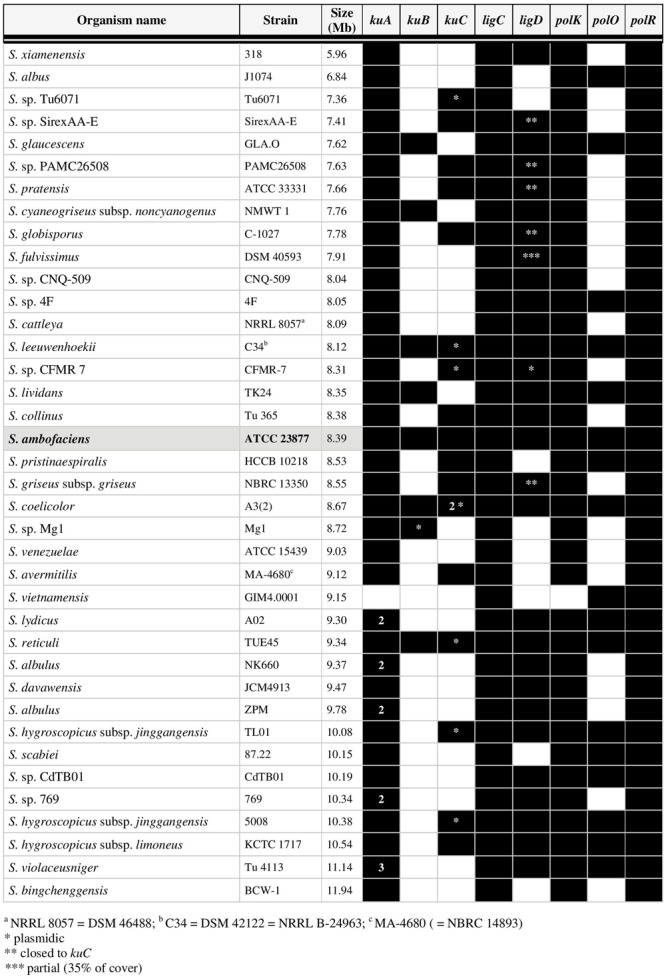
**Distribution of NHEJ-like genes in 38 complete genomes of *Streptomyces*.** The presence of a homolog is mentioned by a black box and the absence of a homolog by an empty box. The number inside the boxes indicates when several homologs were identified; ^∗^ plasmidic; ^∗∗^ closed to *kuC*; *^∗∗∗^* partial (cover of 35%).

Once this list of genes potentially involved in a *Streptomyces* NHEJ pathway established, the question of their conservation in the *Streptomyces* genus was addressed by seeking their homologues in 37 other *Streptomyces* complete genome sequences available at http://www.ncbi.nlm.nih.gov/genome. Therefore, *S. ambofaciens* KuA, KuB, KuC, LigC, LigD, PolK, PolO and PolR were used as query in BLASTP searches. A wide variety of situations were encountered and a few of them are illustrated in **Figure 1**. The homologues identified in 38 *Streptomyces* genomes are listed by their locus tag in Supplementary Table S1 and their presence or absence is summarized in **Figure 2**. The number and position of NHEJ-like genes varied from one species to another. However, two loci are widely shared among *Streptomyces*: *ligC-polR* which is present in all the investigated genomes and *kuA-polK* which is present in all the tested strains but *Streptomyces vietnamensis*.

Focusing on the main function expected from a functional NHEJ system, we analyzed further the Ku-like and LigDom-like encoding genes and extended our interest to the PolDom-like genes colocalizing with them.

**FIGURE 3:**
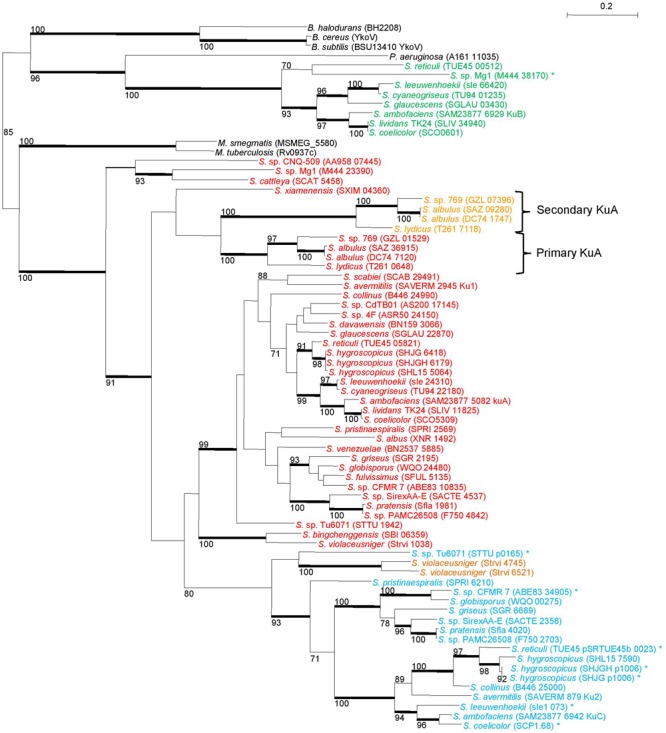
**Phylogeny of Ku-like proteins identified in 38 *Streptomyces* strains.** *Streptomyces* Ku-like proteins were identified in 38 *Streptomyces* strains and compared with the closest homologues of *M. tuberculosis, M. smegmatis, B. subtilis, B cereus, B halodurans*, and *P. aeruginosa*. The tree was built using a maximum-likelihood method with a 295 aa alignment. Only bootstrap values higher than 70% are reported. Heavy lines indicate branches supported by bootstrap values >90% (100 replicates). The scale represents mutations per amino-acid. Primary KuA are in red, secondary KuA are in orange, KuB are in green and KuC in blue. The asterisks symbolized the proteins encoded by plasmid borne-genes. The locus-tag of each sequence is mentioned in brackets. The suspected event of gene duplication is depicted by the two braces. B, *Bacillus*; M, *Mycobacterium*; P, *Pseudomonas*; S, *Streptomyces*. NRRL 8057 = DSM 46488; C34 = DSM 42122 = NRRL B-24963; MA-4680 = NBRC 14893.

All but *S. vietnamensis* possess at least one *kuA* gene. In contrast, *kuB* was detected in only 8 out of the 38 analyzed genomes. Regarding *kuC*, the copy number varies from 0 to 2. Indeed, in the case of *S. coelicolor*, two identical copies of *kuC* are included in the terminal inverted repeats of the SCP1 linear plasmid. A few *Streptomyces* strains carry several copies of *kuA* genes leading us to make a distinction according to their vicinity of *polK*: the neighbors of *polK* were called *kuA* primary genes while standalone *kuA* genes were called secondary *kuA* genes. All the *ku*-like genes (43 *kuA*, 8 *kuB* and 17 *kuC*) were aligned and submitted to a Maximum Likelihood phylogenetic analysis. The tree topology (**Figure 3**), together with the fact that the primary *kuA* gene is almost ubiquitous, suggested that KuA was probably present in the *Streptomyces* common ancestor and evolved along with *Streptomyces*. The secondary *kuA* gene carried by *Streptomyces sp.* 769, *S. albulus* ZPM, *S. albulus* NK660, and *S. lydicus* shares an average nucleotidic identity ranging from 67 to 72% with their respective primary copy. For these species, the primary and secondary KuA phylogenies are congruent, suggesting that their common ancestor gained a second *kuA* copy either by gene duplication or by gene transfer. Regarding the *kuC* gene, it was present in 17 out of 38 analyzed genomes, 7 of which were plasmid-borne. It was closely related to KuA and formed a sister group of KuA within a large KuA-KuC monophyletic group that excluded KuB. In fact *kuB* genes formed a strong monophyletic group and appeared much more scattered in *Streptomyces* genera.

Regarding the distribution of LigC and LigD ligases, LigC encoding genes were found in all genomes and always in the close vicinity of *polR* as in *M. tuberculosis* (**Figure 1**). In contrast, LigD was detected in 29 out of the 38 cases (**Figure 2**). In 6 of them the encoding gene colocalized with *kuC*, and in *S. cattleya*, it colocalized with *kuA*-*polK* (see **Figure 1**). Phylogenetic analysis by Maximum Likelihood method (Supplementary Figure S3) showed that the LigC proteins branched together with *M. tuberculosis* LigC with a topology that seemed quite congruent with the species relations, suggesting a vertical inheritance of this conserved locus. LigD proteins also branched together and together with LigDom of *M. tuberculosis* LigD.

Altogether, two NHEJ-like gene categories were delineated: (i) those that are conserved among the *Streptomyces* and were considered as “core” putative NHEJ factors (i.e., *kuA* and the colocalized *polK, ligC* and the colocalized *polR*) and (ii) those that are sporadically represented among the *Streptomyces* and were considered as “variable” putative NHEJ factors (i.e., *kuB, kuC* and *ligD*).

### NHEJ-Like Genes Are Not Essential in *S. ambofaciens*

After *in silico* identification of the “core” and “variable” NHEJ putative factors in *Streptomyces*, we constructed deleted mutant strains by replacing the chromosomal locus with an excisionable antibiotic resistance cassette (Raynal et al., 2006) by the use of PCR targeting (Gust et al., 2003). The whole selection procedure was performed several times simultaneously in order to select at least two independent mutant lineages for each genetic context. **Table 1** summarizes the different genotypes constructed for this study. The mutations were checked by PCR amplification of the mutated locus. The maintenance of the chromosomal linearity of the mutants was checked by PCR amplification of the telomeres and of some loci scattered along the chromosome (not shown).

All the mutations, simple or multiple, were obtained with classical frequencies indicating that none of the targeted genes (“core” or “variable”) was essential. In addition, there was no significant difference between the wild-type reference strain and any of mutants in colonial morphology and sporulation under normal growth conditions (not shown).

### Implication of NHEJ-Like Genes in DNA Damage Response

**FIGURE 4:**
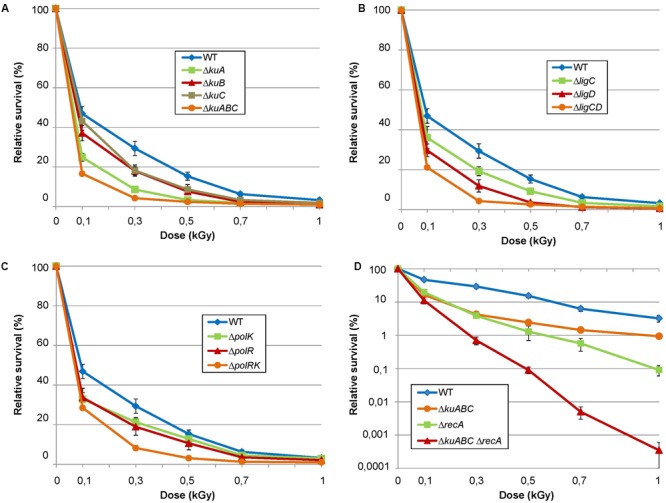
**Electronic beam irradiation of *S. ambofaciens* strains deficient in NHEJ-like genes.** Spores of mutants of NHEJ-like genes, namely single and multiple *ku* **(A)**, ligase **(B)** and polymerase **(C)** mutants, were exposed to 0.1, 0.3, 0.5, 0.7, and 1 kGy of electronic beam irradiation. **(D)** In the same way, a *Δku*-like *ΔrecA* mutant also affected in HR was exposed to the same irradiation doses. In this latter case, a logarithmic scale was used for survival rate representation. Survival rates were calculated as the number of clones for a given dose relative to the number of clones unexposed to radiation. For each graph, wild-type strain is represented as a control. Each curve corresponds to data obtained from an individual mutant strain. Error bars indicate standard error of a minimum of three replicates.

Electronic beam irradiation (EBI) was shown to induce loss of viability of *Bacillus* spores by damaging spore coat, by altering membrane permeability or by DNA fragmentation (Fiester et al., 2012). High level energy beam such as those used in this work (2.2 MeV) is likely to trigger DSB as the most significant lesions in the spore embedded DNA. In order to test the involvement of the NHEJ-like factors to DNA damage response, spores of the wild-type *S. ambofaciens* reference strain as well as spores of at least two independent mutants for each genetic context were irradiated by EBI from 0.1 to 1 kGy and their survival was assessed after growth on SFM medium. The results are represented in **Figure 4**.

#### Both “Core” and “Variable” *ku* genes Are Involved in DNA Damage Response

Depending on the dose, deletion of *kuA*, which belongs to the “core” NHEJ-like gene set, conferred a 2–4 fold increase of the sensitivity compared to the wild-type (**Figure 4A**). Since *kuA* is present in the large majority of the sequenced genome, it appears as the major Ku actor of the putative *Streptomyces* NHEJ. Surprisingly, mutations of the variable *ku*-like genes, *kuB* and *kuC*, also conferred an increased sensitivity but at a lower level, with a maximal twofold cell viability decrease reached after 0.5 and 0.7 kGy exposition for both contexts (**Figure 4A**). In addition, the sensitivity was aggravated when *kuB* and *kuC* were both deleted in a Δ*kuA* background to generate a triple Δ*kuABC* mutant, until reaching a sevenfold cell viability diminution in comparison to the WT for the medium radiation doses (**Figure 4A**). This result suggests that the three Ku products participate to the response to DNA damaging agents in spores, each of them assuming their share of the DNA repair independently from the two others. Additionally, the triple Δ*kuABC* mutation assumed to abolish any putative NHEJ repair strongly increased the sensitivity of a HR deficient context (i.e., Δ*recA*) (**Figure 4D**). This last result shows that RecA and Ku-like proteins are both important contributors to the *S. ambofaciens* spore resistance to EBI.

#### Two ATP Dependent Ligases Involved in DNA Damage Response

In *Mycobacterium*, LigD is the main NHEJ ligase whereas LigC only provides a back-up activity (Della et al., 2004). In contrast to *Mycobacterium* where a single *ligC* mutation does not impact survival rate to DNA damage, the defect of both the conserved *ligC* and the variable *ligD* of *S. ambofaciens* conferred a significant sensitivity to EBI. Surprisingly, the deletion of *ligD* conferred an even higher sensitivity than the mutation of *ligC* (**Figure 4B**). Furthermore, the *ΔligCD* double mutant showed an aggravated phenotype in the same proportions as a *ΔkuABC* triple mutant (**Figure 4B**). These data clearly show that the two ligase activities participate to DNA repair in response to EBI.

#### PolDom Genes Are Involved in DNA Damage Response

Single deletion of *polK* or *polR*, the two conserved homologues of *Mycobacterium* PolDom colocalized respectively with *kuA* and *ligC* led to a weak sensitivity to electronic beam exposure. For both single mutants, increase of sensitivity in comparison to the wild-type does not exceed 1.5 fold for each dose (**Figure 4C**). On the other hand, double *polRK* mutant strains are approximately twice more sensitive than the single mutants (**Figure 4C**). Both loci are therefore involved in DNA damage response.

### The *ku* Genes Are Differentially Expressed during Development

**FIGURE 5:**
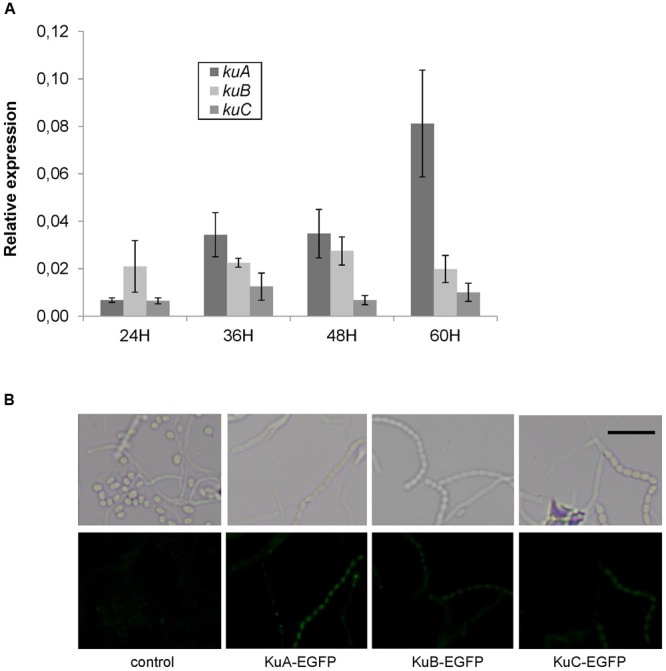
**Growth-dependent and cell-type specific expression of NHEJ-like genes. (A)** Expression level of *S. ambofaciens* ATCC23877 *ku* genes relative to *hrdB* after 24, 36, 48, and 60 h growth on SFM plates at 30°C. Error bars represent standard error. Expression of *kuA* after 60 h growth is significantly higher than that at 24 h. Furthermore, kuA expression at 60 h growth is significantly different than that of *kuB* and *kuC* (*t*-test with Bonferroni correction, α = 0.05). **(B)** Strains expressing EGFP fusion with KuA, KuB, or KuC were inoculated in the acute-angle junction of standard-sized microscope coverslips inserted at 45° in SFM agar, and grown for 48 h before observations by fluorescent microscopy. The control strain does not contain any GFP gene. Representative examples of spores or aerial mycelium are shown as a contrast phase image (upper panels) and as EGFP fluorescent channel (lower panels). The weak fluorescent foci observed mainly in mycelia were readily detected in the control strain and are not likely to correspond to GFP signal. The scale is represented by a 5 μm size bar.

In order to investigate the spatial and temporal expression of NHEJ-like genes during development, a time-course of RT-PCR experiments was performed and translational fusions with EGFP were observed by fluorescent microscopy. First of all, the results showed that NHEJ-like genes are weakly expressed throughout the development. For instance, the expression of *ligC, ligD, polK* and *polR* was too low to be monitored by qPCR whereas the expression of *ku* genes was 10 to 50 fold lower than *hrdB* expression (**Figure 5A**). Secondly, *kuA* expression was clearly growth-phase dependent with a 10 fold increase between 24 and 60 h after germination. It is important to indicate that sporulation began to be observable after 48 h of development in these growth conditions. There was no difference throughout the time-course for *kuB* and *kuC* expression. Both of them were less expressed than *kuA* at the late stages of development. The WT strain was also grown to OD600 of 0.2 in HT liquid medium and then exposed to MMC (1.5 μg⋅ml^-1^) for 30 min. RNAs were extracted and reverse-transcribed to assess by PCR the regulation of NHEJ-like genes during a genotoxic stress. No induction or repression of NHEJ-like genes was noticeable, while the positive induction control (*recA*) was up-regulated up to 20 fold (not shown).

To observe the cellular localization of Ku proteins in physiological conditions, *egfp* translational fusions were constructed at *kuA, kuB*, and *kuC* chromosomal loci. Fusion strains were inoculated on SFM agar plates and grown for 48 h before observations by fluorescent microscopy along with a negative control strain without any *egfp* gene. A positive control strain constitutively expressing *egfp* gene exhibited a strong green fluorescence signal in all cells throughout the developmental stages of the bacteria, proving the reporter gene to be functional in *S. ambofaciens* (not shown). For KuA fusion, the green fluorescence signal was significant in spores and segmenting aerial hyphae but not in vegetative mycelium (**Figure 5B**), showing a specific accumulation of KuA in spores. This result corroborates the phase dependent expression pattern of *kuA* observed in the previous qPCR analysis. For KuB and KuC fusions, although the signals were weaker than for KuA fusion, they also revealed the accumulation of KuB and KuC in spore chains. Since no significant modulation of gene expression was detected for *kuB* and *kuC* along the growth cycle (**Figure 5A**), this protein accumulation pattern suggests a translational (and/or a post-translational) regulation level.

### The Ku Proteins Protect Linear dsDNA from the T5 Exonuclease and Stimulate the Ligase Activity of LigD*_Bsub_*

To assess which proteins are required in a putative *S. ambofaciens* NHEJ, we tried to purify all the proteins putatively implicated in this pathway. Among them, we succeeded to express and purified *S. ambofaciens* KuA, KuB and KuC proteins (39.1, 42.4, and 33.6 KDa respectively). To evaluate the oligomeric state of the three Ku proteins, we performed gel filtration experiments. KuA and KuB were eluted in fractions corresponding to a molecular weight of 160 and 100 kDa, respectively, strongly suggesting that KuA and KuB are multimeric (Supplementary Figure S1 and Supplementary Data Sheet S1). To date, characterized bacterial Ku are dimers. However, the elongated shape of the *B. subtilis* Ku (Ku*_Bsub_*) dimer (McGovern et al., 2016) led to the elution of this protein with apparent molecular weight corresponding to a tetramer. Then KuA and KuB could also be dimers displaying elongated shape, as for Ku*_Bsub_*. KuC was clearly purified in different oligomeric states ranging from 70 to more than 600 kDa. The main elution peak, corresponding to a protein of apparent molecular weight around 160 kDa, was similar to the one observed for KuA.

**FIGURE 6:**
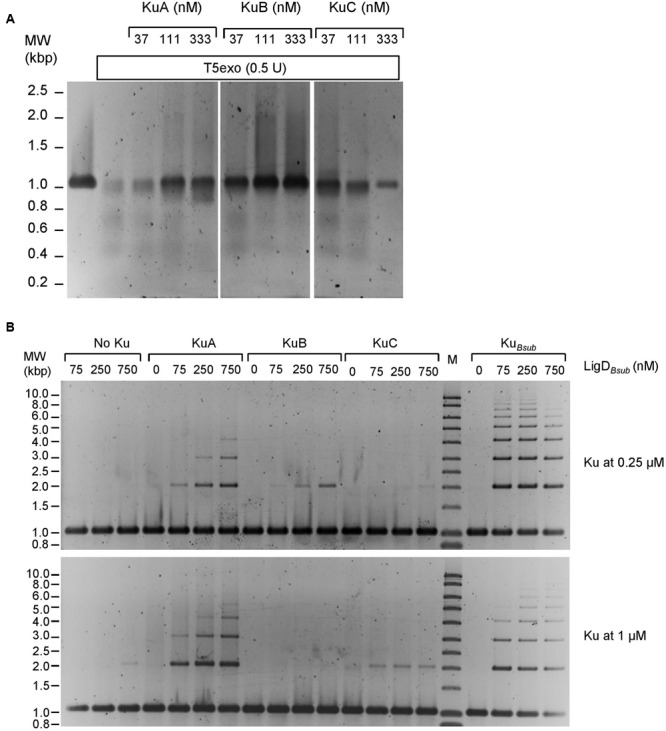
**KuA, KuB, and KuC protect linear dsDNA from the T5 exonuclease and stimulate the ligase activity of LigD*_Bsub_*. (A)** A 1001 bp linear dsDNA with 5′-phosphorylated ends (3.8 nM) was incubated 30 min at 37°C with increasing amounts of KuA, KuB, or KuC as indicated. T5 exonuclease (0.5 U) was then added in each reaction mixtures for 30 min at 37°C and digested products were analyzed as described in the Section “Material and Methods.” Control experiments showing the 1001 ds DNA substrate and the nearly complete degradation of the DNA molecule by the T5 exonuclease without other proteins added are shown on the left. **(B)** A 1001 bp linear dsDNA with 5′-phosphorylated ends (3.8 nM) was incubated with 0.25 or 1 μM of KuA, KuB, or KuC as indicated. Increasing amounts of LigD*_Bsub_* were then added (allowing final concentrations of LigD*_Bsub_* indicated above the gels) and ligation products were analyzed as described in Section “Material and Methods.” Control experiments showing the weak activity of LigD*_Bsub_* alone at different concentration are shown on the left (No Ku). For comparison, Ku*_Bsub_* was assessed for its LigD*_Bsub_* stimulation activity in the same conditions (right part).

KuA, KuB, and KuC were able to protect a linear dsDNA molecule from the T5 exonuclease (**Figure 6A**), a property shared by the Ku*_Bsub_* protein (McGovern et al., 2016) and the *Pseudomonas aeruginosa* Ku against different exonucleases (Zhu and Shuman, 2010). This result suggests that these proteins are able to bind DNA ends. Another role of Ku in the bacterial NHEJ is to recruit the LigD at DNA ends and to stimulate its ligase activity. Remarkably, KuA, KuB, and KuC were able to stimulate the ligation activity of the *B. subtilis* LigD protein (**Figure 6B**), even if they were less efficient than Ku*_Bsub_*. However, we observed a different concentration-dependent stimulation of the ligase by the *S. ambofaciens* Ku proteins. Raising the concentration of KuA and KuC from 0.25 to 1 μM in the ligation assay seemed to slightly increase ligation efficiency. At the opposite, the stimulation activity observed with 0.25 μM of KuB appeared to be reduced when its concentration was raised to 1 μM. A similar inhibitory effect was observed with Ku*_Bsub_*.

## Discussion

### *Streptomyces* NHEJ-Like Gene Organization Is Atypical

The presence of a NHEJ pathway in prokaryotes appears to be limited to some phyla representing about 20% of the species (estimated on a sample of 2645 complete bacterial genomes) (McGovern et al., 2016). The minimal set of NHEJ gene encodes a two partner system including the homodimeric Ku protein and a ligase polypeptide composed of the ligase enzymatic domain (LigDom) to a nuclease (NucDom) and a polymerase (PolDom) domain. The latter enzymatic activities process DNA ends before ligation. The NHEJ gene repertoire ranges from the simplest in *P. aeruginosa* with a single Ku and a ligase (ligase D) to the more complex with those described in *Agrobacterium* (Zhu and Shuman, 2007) or *Sinorhizobium* (Kobayashi et al., 2008) and *Streptomyces* as described in this work. Hence, while *B. subtilis* and *Mycobacterium* possess a single Ku, a single LigD and one or two LigC, *A. tumefaciens* possesses three Ku, two LigD and three LigC (Zhu and Shuman, 2007). *Sinorhizobium meliloti* and other rhizobia possess up to 4 Ku, 5 LigD and one LigC (Kobayashi et al., 2008); the ligase, nuclease and polymerase domains are present in different organizations, sometimes (i) fused together in a single polypeptide or (ii) encoded by distinct genes. This situation is similar in archaea where the putative ligase, nuclease and polymerase domains are encoded by distinct genes, however, kept into an operonic organization assumed to ensure the co-regulation of the activities (Bartlett et al., 2013). However, among actinobacteria, *Streptomyces* appears as an exception, since other main phyla (*Nocardia, Frankia, Rhodococcus*, and *Corynebacterium*) seem to possess a multidomain LigD homologue (not shown).

In *Streptomyces*, several levels of complexity are also noticeable. First, the activities putatively involved in NHEJ are encoded by standalone genes that are scattered along the genome. Consequently the repertoire is rather expanded: the number of genes ranging from none up to 4 for *ku*, from one to two LigDom encoding genes (*ligC, ligD*), from 2 to 3 PolDom encoding genes, from none to one NucDom encoding gene. The total number of NHEJ-like genes ranges from 4 in *S. bingchenggensis* and *S. venezuelae* to 8 in *S. ambofaciens* and *S. reticuli*: 3 *ku* (*kuA, kuB, kuC*), one *ligD* and one *ligC*, and 3 polymerase genes (*polK, polR, polO*). These genes can fall into two groups. On one hand, the “core” set includes *kuA, ligC, polK* and *polR* which are present in almost all *Streptomyces* species and organized in two loci of rather conserved organization (i.e., *kuA*-*polK, ligC*-*polR*). The only exception appears to be in *S. vietnamiensis* which lacks the couple *kuA*-*polK* and the other *ku* genes. On the other hand, the rest of the genes constitute the “variable” gene set. An interesting feature to highlight is a gene duplication event involving *kuA* or *kuC*. The duplicated copies are either located in the variable region of the genome or present within the terminal inverted repeats of the linear replicons. This phenomenon is interesting since duplicated copies can evolve through gene divergence to raise new function. This mechanism is rather rare in bacteria but gene duplication seems frequent in *Streptomyces*, notably with large terminal duplication probably resulting from DSB repair by a mechanism related to break-induced replication.

### Involvement of the “Core” and “Variable” NHEJ-Like Gene Sets in DNA Repair

The large number of NHEJ-like genes in *Streptomyces* as well as their variability questions their implication in DNA repair mechanisms. It is tempting to imply the “core” NHEJ gene set in a classical NHEJ pathway with a Ku (i.e., KuA) and an ATP-dependent DNA ligase (i.e., LigC). In addition to being the conserved *ku* gene, *kuA* is the more expressed of the 3 *ku-*like genes in the late growth phases, its defect conferred the most marked sensitivity to EBI and finally, KuA is the most efficient of the 3 Ku-like proteins in stimulating LigD*_Bsub_* activity *in vitro*.

It might be surprising that the NHEJ ligase could be LigC rather than LigD which is the canonical NHEJ ligase in other bacterial species. However, it should be reminded that in *Streptomyces*, LigD carries the only ligase domain, as does LigC. Both ligases are devoid of the PolDom domain known to be involved in its recruitment by Ku. Therefore LigD and LigC may ensure similar functional roles in *Streptomyces*. One could hypothesize that a gene rearrangement split the ligase domain from the two others (i.e., PolDom and NucDom) in a *Streptomyces* common ancestor. The two ligase genes would have then provided an equivalent contribution to the bacterium, finally resulting in the replacement of LigD by LigC in the “core” NHEJ set by a founder effect.

In *S. ambofaciens*, in which both *ligC* and *ligD* cohabits, single mutants displayed a sensitive phenotype to EBI and the double mutant displayed an aggravated phenotype. In this context, at least two hypotheses can be drawn. First, both LigC and LigD could participate to the same repair pathway; in that case, the sensitivity of single mutants and the aggravated phenotype of double mutants could be explained by a dose effect that is the need for a certain amount of ligases to cope for the induced DNA damage. In that case, both ligase genes would contribute to achieve the required level of ligase. Alternatively, they could participate to different repair pathways required to take over varied DNA damage (see below).

If the “core” NHEJ gene set indeed establishes a functional NHEJ pathway, the presence of two PolDoms in the “core” NHEJ gene set constitutes another intriguing feature, especially considering that they do not seem to have redundant functions. Hence, the sensitivity of both *polK* and *polR* simple mutants was aggravated in the double mutant. As shown in *M. tuberculosis* NHEJ, the role of the PolDom domain of LigD would be less to provide a polymerase activity than to recruit LigD to Ku (Pitcher et al., 2005). Then PolK and/or PolR may play a role of linker/adapter to recruit a ligase; the more likely being that PolK and PolR recruit LigC, however, on different types of DNA extremities (e.g., blunt, 3′ or 5′ overhanging ends). In the case of the presence of LigD (as in *S. ambofaciens* for example), the two PolK and PolR may recruit both LigC and LigD.

It is particularly noticeable that the “variable” genes (i.e., *ligD* but also *kuB, kuC*) were involved in the response to EBI. The exposure to EBI is assumed to trigger DSBs. However, different types of lesions can result from direct transmission of energy to the DNA structure and from secondary oxidative stress (e.g., oxidized bases). In consequence, damage induced by EBI requires NHEJ but may also require other DNA repair pathways. Thus, these “variable” genes would (i) support the NHEJ encoded by the “core” gene set in an opportunistic way, (ii) constitute an alternative NHEJ pathways or (iii) constitute a repair pathway dedicated to other DNA damage single strand gaps or abasic sites). The first hypothesis would imply a dose effect of the Ku as well as the ligase proteins to explain the aggravated phenotype of the multiple mutants (i.e., *ΔkuABC* and *ΔligCD*). In this first hypothesis, considering that prokaryote Ku act as dimers (McGovern et al., 2016), we can speculate that these three Ku proteins could act as homodimers as well as heterodimers. A data in favour of the second hypothesis (an alternative NHEJ pathway) is the colocalization of the *kuC* and *ligD* genes in 6 *Streptomyces* species clustering with *S. griseus* (Supplementary Table S1; **Figure 3**). However, no clear trend toward the co-occurrence of these two “variable” genes can be noticed in the 38 analysed genomes. In *B. subtilis*, the recent involvement of LigD*_Bsub_* and Ku*_Bsub_* in base excision repair along with their major role in NHEJ supports the third hypothesis (de Ory et al., 2014, 2016).

In order to solve the question of the involvement of these genes in a NHEJ pathway, a forward-looking approach could be to specifically induce DSB formation at targeted chromosomal loci and assessed the sensitivity of the NHEJ-candidate genes to identify those truly involved in DSB repair *per se*.

### A Late Growth Phase Role for NHEJ in *Streptomyces*

The presence of a NHEJ pathway in bacteria is usually assigned to DSB repair in low rate growth phases characterizing bacterial species involved in pathogenic processes or exposed to harsh environmental living conditions. When bacteria replicate such as in laboratory conditions, sister chromosomes providing an intact template are preferentially used to ensure faithful DSB repair by HR. However, when replication is slowing down (i.e., stationary phases) or stopping (e.g., in resistance forms such as spores), DSB repair strongly relies on NHEJ. This is consistent with the fact that NHEJ genes are expressed preferentially when DSB repair cannot be ensured by a faithful process. Hence, in *B. subtilis* as well as in *Streptomyces*, the NHEJ genes were shown to be expressed in stationary phase and to accumulate in spores. The forespore-specific sigma factor σ^G^ in *B. subtilis* directly regulates *ykoU* (coding for LigD) and *ykoV* (coding for Ku), inducing their expression during the forespore development (Wang et al., 2006). In addition, *B. subtilis* Ku protein specifically localizes to the nucleoid of germinating spores while it disappears at later stages of germination. In *Streptomyces*, gene transcription analyses showed in this work an expression of the *ku* in stationary phase with an increase of *kuA* expression during sporulation. Moreover, GFP fusions showed a predominant localization of the Ku proteins in spores. Furthermore, mutations in *ku* and ligase genes confer sensitivity to genotoxic agents to *B. subtilis* as well as *Streptomyces* spores. Sensitivity to heat and desiccation which are biotic parameters known to trigger DSB is also associated to the NHEJ mutations in *B. subtilis* and *Mycobacterium smegmatis* (Moeller et al., 2007; Pitcher et al., 2007; Stephanou et al., 2007). The presence of multiple *ku* genes (and of other NHEJ-like genes) in *Sinorhizobium meliloti* is assumed to correspond to the need for DNA repair at different steps of the cell cycle. Although no induction of the NHEJ pathways was observed under genotoxic conditions (i.e., IR exposure), the expression patterns of the *sku* genes suggest that each Ku protein has a distinct role under different conditions (free bacterial form, bacteroids). In contrast, NHEJ would not be required during the establishment of symbiosis since a strain lacking all four *sku* genes was shown to establish functional nodules (Kobayashi et al., 2008). As in *S. meliloti*, it was shown that each of the *S. ambofaciens ku* genes is functional since their deletion conferred a phenotype of sensitivity toward genotoxic agents. Mutant strains lacking *kuA, kuB*, and *kuC* genes showed an aggravated phenotype suggesting that each participated to the response to DNA damage. We can therefore suppose that like in *S. meliloti*, intervention of the *Streptomyces ku* genes is done under specific conditions.

Recently in *Pseudomonas putida*, the NHEJ was shown to participate to another late growth phase phenomenon (Paris et al., 2015). Hence, bacteria frequently encountered nutrient starvation during late-growth phase. Populations starving display a phenomenon called stationary-phase mutagenesis (also known as adaptive mutations) from which emerge mutants able to overcome the shortage and initiate new vegetative cycles. The underlying mechanisms notably involve unfaithful DNA polymerases during HR events (Shee et al., 2012). Although the involvement of NHEJ in late mutagenesis remains unknown, deficiency of each of the NHEJ activities triggered an altered pattern of mutations (Paris et al., 2015). The authors speculate on ability to bypass DNA lesions of the LigD polymerase domain.

### Origin of the “Core” and “Variable” NHEJ Gene Sets in *Streptomyces*

While the origin of the NHEJ “core” gene set is likely to result from vertical inheritance from a common *Streptomyces* ancestor, the origin of the “variable” set is questionable. The sporadic distribution of the “variable” genes may reflect either the existence of NHEJ in a bacterial common ancestor and its subsequent sporadic loss during genus/species separation. Reciprocally, it may reflect the acquisition of NHEJ genes through horizontal gene transfer in some phyla. Both hypotheses can also be combined and result in the observed complexity. The most explicit argument about their origin is their localization in the variable regions of the chromosome or in plasmids. While plasmids can be mobilized and even self-transmitted, the terminal regions of the *Streptomyces* chromosome is known to be targeted by recombination phenomena leading to insertions of acquired DNA and further losses (Choulet et al., 2006).

### Impact of NHEJ on Genome Evolution

Whatever the evolutionary past of the NHEJ gene sets, the presence of a conserved “core” gene set enriched by additional functional “variable” genes may confer some advantage. In all bacteria studied so far, NHEJ is not essential (Ku/LigD) for growth, at least under laboratory conditions. Mutants of NHEJ in *Mycobacterium, Pseudomonas*, or *Bacillus* are not affected although they show sensitivity to environmental stress (Weller et al., 2002; Jacobs et al., 2003; Gong et al., 2004, 2005). We came to the same conclusion in *S. ambofaciens* although it was particularly interesting to note that a part of the “variable” genes (i.e., *kuB, kuC* and *ligD*) was involved in response to genotoxic agents. Hence the aggravated phenotype observed in double or multiple mutant strains (i.e., *kuABC, ligCD*) revealed the cooperative actions of the multiple alleles. The “variable” genes in *Streptomyces* may increase the flexibility and/or efficiency of the DSB repair mechanism and influence evolution of the genome. Lessons about the role of NHEJ in genome evolution can be drawn from organisms lacking NHEJ. Hence, the only eukaryote lacking NHEJ is the yeast *Lachancea kluyveri* (Gordon et al., 2011). Compared to other species of the Saccharomycetaceae family, this species showed a significantly low number of genomic rearrangements and the lack of telomere-to-telomere fusions. The authors suggest that the loss of the NHEJ and an alternative end joining pathway [named microhomology-mediated end joining (MMEJ) or alternative end joining (A-EJ)] is responsible for the low recombination rate assessed in this phylum. In this way, we can hypothesize that in *Streptomyces* the presence/absence of NHEJ genes in addition to the “core” NHEJ (the “variable” genes participating to a unique NHEJ or other alternative pathways) may modulate the frequency of DNA rearrangements and DNA acquisition through horizontal gene transfer and confer different evolution rates to *Streptomyces* species. These genes being themselves subject to transfer, the recombinant phenotype (i.e., hyperrecombinant) could be variable within a population and favor genetic diversification and adaptation to the environment. This situation could be reminiscent of mutator strains identified in *E. coli* (for review: Giraud et al., 2001) but applied to the capacity to recombine DNA including incoming genetic information.

### Future Perspectives

A key question is the relative contribution of HR and NHEJ to the evolution and the remarkable compartmentalization of the linear *Streptomyces* chromosome. Is there any regulation between the two pathways during the development of the bacteria? In *S. avermitilis*, the deletion of the *ku* homologues (*ku1* and *ku2* corresponding to *kuA* and *kuC* respectively) was associated to an increased efficiency of HR (Zhang et al., 2012). This may reveal the existence of a balance resulting from the competition between HR and NHEJ repair mechanisms, and that both repair pathways co-exist at least during replicating phases. Is there any spatial regulation of the DSB repair mechanisms? DSB could be healed by different mechanisms depending of their chromosomal location leading to either faithful repair or possible integration of foreign information.

## Author Contributions

Investigation: GH, CB, LZ, EP, LC, and SM; Computer analysis: AT and CyB; Methodology: CyB, FC, and FL; Conceptualization: CB, AT, FL, and PL; Writing original draft: GH, CB, AT, and PL; Writing-review and editing: FL, FC, CyB, CB, AT, and PL; Resources: FC, FL, and PL; Funding acquisition: CB, AT, and PL; Supervision: AT and PL.

## Conflict of Interest Statement

The authors declare that the research was conducted in the absence of any commercial or financial relationships that could be construed as a potential conflict of interest.

## Footnotes

1 http://www.ncbi.nlm.nih.gov/assembly/?term=Streptomyces
