# Supplementary material for: Multiple and Variable NHEJ-Like Genes Are Involved in Resistance to DNA Damage in Streptomyces ambofaciens
Source: Front Microbiol. 2016 Nov 28;7:1901. doi: 10.3389/fmicb.2016.01901 (PMC5124664; doi:10.3389/fmicb.2016.01901)
Supplement: Supplementary file 1 [file Table_1.PDF]

## Table S1: Identification of NHEJ-like genes in 38 complete genomes of *Streptomyces* by their locus_tag

| Organism[a] | Strain | Ku-like | | | ATP-dependant DNA ligase | | Poldom-like | | |
|---|---|---|---|---|---|---|---|---|---|
| | | KuA | KuB | KuC | LigC | LigD | PolK | PolO | PolR |
| *S. xiamenensis* | 318 | SXIM_04360 | | - | SXIM_50860 | SXIM_51150 | SXIM_04370 | - | SXIM_50850 |
| *S. albus* | J1074 | XNR_1492 | | - | XNR_0334 | - | XNR_1493 | XNR_4488 | XNR_0333 |
| *S.* sp. Tu6071 | Tu6071 | STTU_1942 | | STTU_p0165 (plasmidic) | STTU_0771 | - | STTU_1943 | - | STTU_0755 |
| *S.* sp. SirexAA-E | SirexAA-E | SACTE_4537 | | SACTE_2358 | SACTE_5876 | SACTE_2357 ** | SACTE_4536 | - | SACTE_5877 |
| *S. glaucescens* | GLA.O | SGLAU_22870 | SGLAU_03430 | - | SGLAU_28040 | SGLAU_02470 | SGLAU_22865 | SGLAU_27360 | SGLAU_28045 |
| *S.* sp. PAMC26508 | PAMC26508 | F750_4842 | | F750_2703 | F750_6167 | F750_2702 ** | F750_4841 | - | F750_6168 |
| *S. pratensis* | ATCC 33331 | Sfla_1981 | | Sfla_4020 | Sfla_0697 | Sfla_4021 ** | Sfla_1982 | | Sfla_0696 |
| *S. cyaneogriseus* | NMWT 1 | TU94_22180 | TU94_01235 | - | TU94_28785 | TU94_01705 | TU94_22175 | | TU94_28790 |
| *S. globisporus* | C-1027 | WQO_24480 | | WQO_00275 | WQO_30485 | WQO_00280 ** | WQO_24475 | - | WQO_30490 |
| *S. fulvissimus* | DSM 40593 | SFUL_5135 | | - | SFUL_6473 | SFUL_6958 partial | SFUL_5134 | - | SFUL_6474 |
| *S.* sp. CNQ-509 | CNQ-509 | AA958_07445 | | - | AA958_31995 | AA958_07435 | AA958_07440 | - | AA958_31990 |
| *S.* sp. 4F | 4F | ASR50_24150 | | - | ASR50_30225 | ASR50_04130 | ASR50_24145 | ASR50_29170 | ASR50_30230 |
| *S. cattleya* | NRRL 8057 | SCAT_5458 | | - | SCAT_5513 | SCAT_5457 | SCAT_5459 | - | SCAT_5514 |
| *S. leeuwenhoekii* | C34 | sle_24310 | sle_66420 | sle1_073 (plasmidic) | sle_11180 | sle_65450 | sle_24320 | sle_12820 | sle_11170 |
| *S.* sp. CFMR 7 | CFMR-7 | ABE83_10835 | | ABE83_34905 (plasmidic) | ABE83_03950 | ABE83_34900 (plasmidic) ** | ABE83_10840 | - | ABE83_03945 |
| *S. lividans* | TK24 | SLIV_11825 | SLIV_34940 | - | SLIV_04970 | SLIV_02570 | SLIV_11830 | SLIV_05935 | SLIV_04965 |
| *S. collinus* | Tu 365 | B446_24990 | | B446_25000 | B446_30620 | B446_04080 | B446_24985 | - | B446_30625 |
| ***S. ambofaciens*** | **ATCC 23877** | **SAM23877_5082** | **SAM23877_6929** | **SAM23877_6942** | **SAM23877_6361** | **SAM23877_0862** | **SAM23877_5081** | **SAM23877_6202** | **SAM23877_6362** |
| *S. pristinaespiralis* | HCCB 10218 | SPRI_2569 | | SPRI_6210 | SPRI_1068 | - | SPRI_2570 | SPRI_6912 | SPRI_1067 |
| *S. griseus* | NBRC 13350 | SGR_2195 | | SGR_6689 | SGR_1024 | SGR_6690 ** | SGR_2196 | - | SGR_1023 |
| *S. coelicolor* | A3(2) | SCO5309 | SCO0601 | SCP1.68 (plasmidic) | SCO6707 | SCO7345 | SCO5308 | SCO6498 | SCO6709 |
| *S.* sp. Mg1 | Mg1 | M444_23390 | M444_38170 (plasmidic) | - | M444_27900 | - | M444_23395 | | M444_27905 |
| *S. venezuelae* | ATCC 15439 | BN2537_5885 | | - | BN2537_3729 | - | BN2537_5887 | - | BN2537_3727 |
| *S. avermitilis* | MA-4680 | SAVERM_2945 | | SAVERM_879 | SAVERM_1697 | - | SAVERM_2946 | - | SAVERM_1696 |
| *S. vietnamensis* | GIM4.0001 | - | | - | SVTN_31170 | - | - | SVTN_30635 | SVTN_31290 |
| *S. lydicus* | A02 | T261_0648 ;<br>T261_7118 | | - | T261_0463 | T261_1796 | T261_0647 | T261_1305 | T261_0462 |
| *S. reticuli* | - | TUE45_05821 | TUE45_00512 | pSRTUE45b_0023 (plasmidic) | TUE45_07256 | TUE45_00866 | TUE45_05820 | TUE45_07099 | TUE45_07257 |
| *S. albulus* | NK660 | DC74_7120 ;<br>DC74_1747 | | - | DC74_7353 | DC74_6447 | DC74_7121 | - | DC74_7354 |
| *S. davawensis* | JCM4913 | BN159_3066 | | - | BN159_1716 | BN159_8336 | BN159_3067 | - | BN159_1715 |
| *S. albulus* | ZPM | SAZ_36915 ;<br>SAZ_09280 | | - | SAZ_38065 | SAZ_33260 | SAZ_36920 | - | SAZ_38070 |
| *S. hygroscopicus* | TL01 | SHJGH_6179 | | SHJGH_p1006 (plasmidic) | SHJGH_7371 | SHJGH_1851 | SHJGH_6178 | SHJGH_7216 | SHJGH_7372 |
| *S. scabiei* | 87.22 | SCAB_29491 | | - | SCAB_13591 | - | SCAB_29521 | SCAB_17401 | SCAB_13581 |
| *S.* sp. CdTB01 | CdTB01 | AS200_17145 | | - | AS200_09240 | AS200_41235 | AS200_17150 | AS200_10695 | AS200_09235 |
| *S.* sp. 769 | 769 | GZL_01529 ;<br>GZL_07396 | | - | GZL_01249 | GZL_02561 | GZL_01528 | - | GZL_01248 |
| *S. hygroscopicus* | 5008 | SHJG_6418 | | SHJG_p1006 (plasmidic) | SHJG_7610 | SHJG_2086 | SHJG_6417 | SHJG_7456 | SHJG_7611 |
| *S. hygroscopicus* | KCTC 1717 | SHL15_5064 | | SHL15_7590 | SHL15_6478 | SHL15_0572 | SHL15_5063 | SHL15_6335 | SHL15_6479 |
| *S. violaceusniger* | Tu 4113 | Strvi_1038 ;<br>Strvi_6521 ;<br>Strvi_4745 | | - | Strvi_3581 | Strvi_5345 | Strvi_1039 | Strvi_0339 | Strvi_3580 |
| *S. bingchenggensis* | BCW-1 | SBI_06359 | | - | SBI_08910 | - | SBI_06360 | - | SBI_08909 |

[a] the species have been ordered by their genome size, from the smaller to the larger.

** *ligD* gene colocalizing with *kuC* gene
