# Supplementary material for: Multiple and Variable NHEJ-Like Genes Are Involved in Resistance to DNA Damage in Streptomyces ambofaciens
Source: Front Microbiol. 2016 Nov 28;7:1901. doi: 10.3389/fmicb.2016.01901 (PMC5124664; doi:10.3389/fmicb.2016.01901)
Supplement: Supplementary file 2 [file Data_Sheet_1.PDF]

SUPPLEMENTARY MATERIAL

Gel filtration assay

Two nmol of KuA, KuB or KuC proteins were incubated in 500 µL of buffer G (50 mM Tris-HCl, pH 8, 250 mM NaCl) for 10 minutes at 4°C. After centrifugation at 13000 rpm, the soluble fraction was injected on a Superdex 200 10/300 GL (GE Healthcare) equilibrated with buffer G. Twenty two fractions of 500 µL were collected, ranging from approximately 700 kDa (the void volume) to 10 kDa. To compare the elution volumes of the proteins, 5 µL of SDS-loading buffer were added to 20 µL of each of these fractions and loaded onto 10 % acrylamide SDS-PAGE gels. After migration, gels were stained with Coomassie blue.
