# Supplementary material for: Multiple and Variable NHEJ-Like Genes Are Involved in Resistance to DNA Damage in Streptomyces ambofaciens
Source: Front Microbiol. 2016 Nov 28;7:1901. doi: 10.3389/fmicb.2016.01901 (PMC5124664; doi:10.3389/fmicb.2016.01901)
Supplement: Supplementary file 3 [file Image_1.PDF]

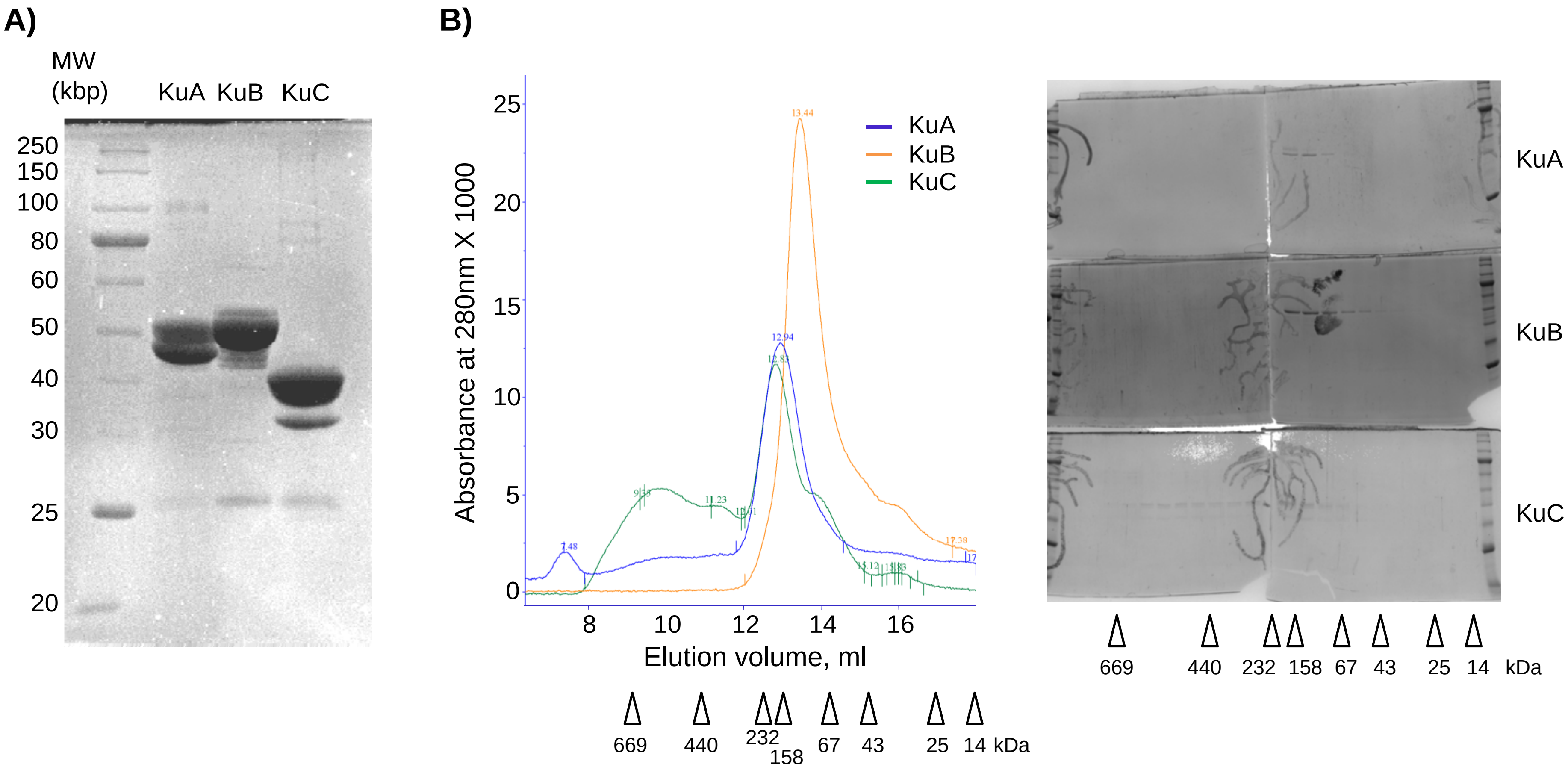

Figure S1: Analysis of the purified KuA, KuB and KuC proteins. **A)** SDS PAGE analysis of purified fractions containing 10 µg of proteins obtained after elution from Ni-NTA agarose and dialysis. **B)** Gel filtration analysis of the purified Ku proteins. Chromatograms are presented on the left and protein contents analyzed by SDS PAGE on the right. Nearly all proteins in the KuA or KuB preparations were eluted in one elution peak corresponding to a molecular weight region around 160 kDa for KuA (39.1 kDa for the monomer) and 100 kDa for KuB (42.4 kDa for the monomer). Main proteins in the fraction containing KuC were co-eluted all along the gel filtration chromatography, from the void volume (molecular weight greater than 700 kD) to elution volumes corresponding to 70 kDa (33.6 kDA for the monomer).
