# Supplementary material for: Multiple and Variable NHEJ-Like Genes Are Involved in Resistance to DNA Damage in Streptomyces ambofaciens
Source: Front Microbiol. 2016 Nov 28;7:1901. doi: 10.3389/fmicb.2016.01901 (PMC5124664; doi:10.3389/fmicb.2016.01901)
Supplement: Supplementary file 4 [file Image_2.PDF]

**Figure S2: Alignment of amino-acid sequences of Ku-like proteins of *S. ambofaciens***

KuA, KuB and KuC proteins of *S. ambofaciens* composed of 365, 383 and 302 amino-acids respectively and the 311 amino-acid Ku$_{Bsub}$ were aligned with ClustalW. The Ku core domain and the C-terminal minimal domain are boxed in grey and red, respectively. The SAP domain previously predicted in the *S. coelicolor* KuB homologue by (Aravind and Koonin 2001) is highlighted in yellow.

```
                                     10        20        30        40        50        60        70        80
                             ....|....|....|....|....|....|....|....|....|....|....|....|....|....|....|....|
B. subtilis (BSU13410 = YkoV) MNRTPSLHTKEKKGFIDMHTMWKGSISFGLVNIPIKLYAATEDKDIKLRSLHKEDHAPIKYEKVCTNCEKTLSPDEIVKG
S. ambofaciens (KuA)          -----------------MRSIWNGAISFGLVSIPIKLMNATESHSVSFRQIHMEDGGRIRYRKVCELEDREVTQDEIGKA
S. ambofaciens (KuB)          ----------------MARAIWTGVITFGLVSVPVGVFTATQDHTVHFHQLQRGTSDRVRNRRVNERTGEEVDAGDIVKG
S. ambofaciens (KuC)          ----------------MPRTIWSGAISFGLVTVPIDVVSATEDHSIHFHRVHLDDMARVRTRKVCELEDREVSSSEIGKG

                                     90       100       110       120       130       140       150       160
                             ....|....|....|....|....|....|....|....|....|....|....|....|....|....|....|....|
B. subtilis (BSU13410 = YkoV  YEYVKGKYVVLTDEDLKSLKQEHEEKAVEIVDFVQLQEIDPIYFNRSYFVGP-GDNGTKAYTLLREALRSTGKIGIANMT
S. ambofaciens (KuA)          YEDADGSMIPITDDDLTQLPIP-TARTIEIVAFVPEERIDPLQMGSAYYLAASGAPAAKPYTLLREALKRSNRVAIAKFA
S. ambofaciens (KuB)          YEVGEGEYVVVQPEELDEIAPG-RSRTLEITDFVDLDRIAPVYFDRTYYVAPRGEEYLKVYELLRTALAETGKAGVATFV
S. ambofaciens (KuC)          YELSRTQVIPISDEELRDLPLP-TAKAIEIVAFMPWESVDPLRIAEGYYLAPDGQVAAKPYKLLRQALERSSRVAVARYA

                                    170       180       190       200       210       220       230       240
                             ....|....|....|....|....|....|....|....|....|....|....|....|....|....|....|....|
B. subtilis (BSU13410 = YkoV) IRSKQQLAILRVYENCIVMESIHYPDEVRSAAQVPGVPDQSNVN-DKELQTAITLIDELTA-KFEPEKYEDTYRQALLQR
S. ambofaciens (KuA)          LRGRERLGMLRVVGDAIAMHGLLWPDEVRAPEGVAPDAGVTVRD--QELDLADALMDTLG--EIDLDDLHDEYREALEEV
S. ambofaciens (KuB)          MRNRQYLTALRAEDEVLVLQTLHWADEVRDPGEELPELPSDRAGRGKELDMALRLVEALSG-PWDPARYRDTYQERVREL
S. ambofaciens (KuC)          WSGRERLGLLRVREEAIVLHAMRWPDEIRDPSSLTPPP-VELTD--EEIDGALALMDTMTRDGLEGEEFRDTYTEAVAEL

                                    250       260       270       280       290       300       310       320
                             ....|....|....|....|....|....|....|....|....|....|....|....|....|....|....|....|
B. subtilis (BSU13410 = YkoV) VNDKLENKETAVTPDKAPPR-EDVIDLVSALQASIDRTR-----------------------------RPNRETPAAAPA
S. ambofaciens (KuA)          IAAKAAG-ETPRESPEPAAP-GKVLDLMAALEGSVRAARESRGGEGAGAAEGAEVRSLPRRA-ASARRTPKETGGKKSTS
S. ambofaciens (KuB)          VRAKAEGTEIAVAEEAPGAT--NVVDLMEALRGSLEQAGGEGGQPPDEGRKGDRSSAARKRSSAEEKRRPGKRASAGRTQ
S. ambofaciens (KuC)          IEAKREHREPPAAKEEPAAESGQLVDLMAALQESVSKAKATRG---------------------------EDTGGHADVH

                                    330       340       350       360       370       380       390       400
                             ....|....|....|....|....|....|....|....|....|....|....|....|....|....|....|....|
B. subtilis (BSU13410 = YkoV) QAAEPKGAGDKKQKTT---------------RKKASGTS-----------------------------------------
S. ambofaciens (KuA)          TAAKKPAAKKAEAKKSTASATGKPAAKSGRAAKKTADPGTA-----------KKATGKGATTRNTASKGTAKRTAPRKRS
S. ambofaciens (KuB)          GSAEKKRATKGKEKESAKRKKAGAPSRRTTPKEKRSDLREMSKAELYRRAGDQGIAGRSRMSRDQLIEALSGTGDRRGRT
S. ambofaciens (KuC)          EMPKKRTAKKGPAKKT--------------AAKKTARK-------------------------------------PR-RS

                              ...
B. subtilis (BSU13410 = YkoV) ---
S. ambofaciens (KuA)          A--
S. ambofaciens (KuB)          AAA
S. ambofaciens (KuC)          A--
```
