# Supplementary material for: Multiple and Variable NHEJ-Like Genes Are Involved in Resistance to DNA Damage in Streptomyces ambofaciens
Source: Front Microbiol. 2016 Nov 28;7:1901. doi: 10.3389/fmicb.2016.01901 (PMC5124664; doi:10.3389/fmicb.2016.01901)
Supplement: Supplementary file 5 [file Image_3.PDF]

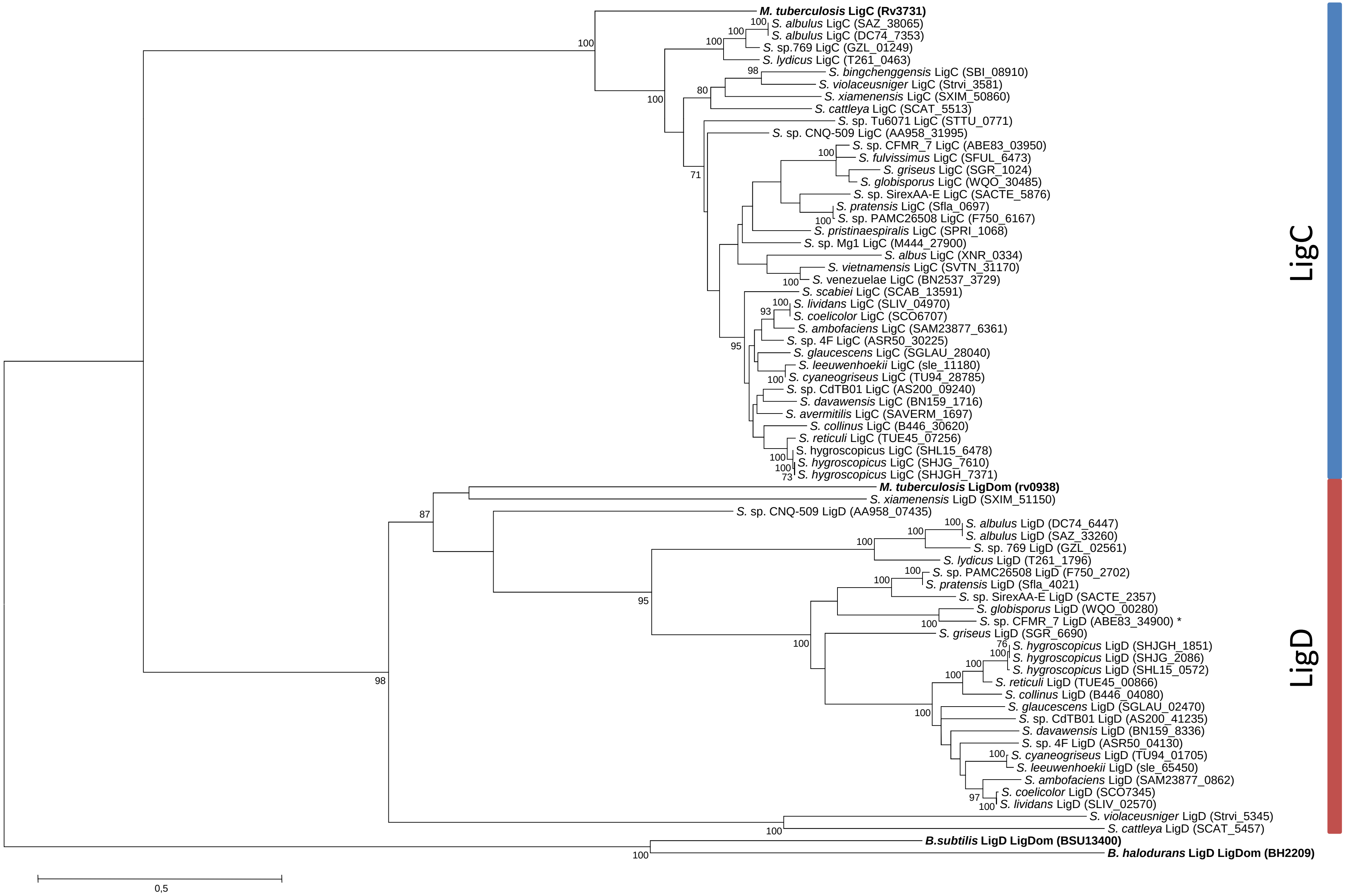


**Figure S3: Phylogeny of *Streptomyces* LigC and LigD proteins.** *Streptomyces* LigC and LigD proteins identified in 38 *Streptomyces* strains were compared with their homologues in *M. tuberculosis*. LigD homologues in *B. subtilis* and *B halodurans* were used as a root. The tree was built using a maximum-likelihood method with a 309 aa position alignment. Numbers indicate by bootstrap values > 70% (100 replicates). The scale represents mutations per amino acid.

The locus-tag of each sequence is mentioned in brackets. B : *Bacillus*, M : *Mycobacterium*, S : *Streptomyces*.
